# Comparative analysis of whole blood transcriptomics between European and local Caribbean pigs in response to feed restriction in a tropical climate

**DOI:** 10.1186/s12864-023-09381-7

**Published:** 2023-05-30

**Authors:** Nausicaa Poullet, Orianne Devarieux, David Beramice, Laurent Dantec, Yoann Félicité, Dalila Feuillet, Jean-Luc Gourdine, Jean-Christophe Bambou

**Affiliations:** 1 ASSET, INRAE, Petit-Bourg (Guadeloupe), ²PTEA, INRAE, Petit-Bourg (Guadeloupe), 97170 France; 2 PTEA, INRAE, Petit-Bourg (Guadeloupe), 97170 France

**Keywords:** Blood transcriptome, Feed restriction, Refeeding, Creole pig, Tropical climate

## Abstract

**Background:**

Feed restriction occurs frequently during pig growth, either due to economic reasons or stressful environmental conditions. Local breeds are suggested to have better tolerance to periods of feed restriction. However, the mechanisms underlying the response to feed restriction in different breeds is largely unknown. The aims of the present study were (1) to compare the blood transcriptome profile in response to feed restriction and refeeding of two contrasted breeds, Large White (LW), which has been selected for high performance, and Creole (CR), which is adapted to tropical conditions, and (2) to investigate the effect of a moderate feed restriction and refeeding on whole blood transcriptome. Analysis of blood transcriptome allows to study the response to feed restriction and refeeding in a dynamic way. RNAseq was performed on blood samples of growing LW and CR pigs at two time points: after 3 weeks of feed restriction and after 3 weeks of refeeding. The data was compared with samples from control animals offered the same diet on an *ad libitum* basis throughout the whole experiment.

**Results:**

In terms of performance (body weight and feed efficiency), CR pigs were less impacted by feed restriction than LW. The transcriptional response to feed restriction and refeeding between CR and LW was contrasted both in terms of number of DEGs and enriched pathways. CR demonstrated a stronger transcriptional response to feed restriction whereas LW had a stronger response to refeeding. Differences in the transcriptional response to feed restriction between CR and LW were related to cell stress response (Aldosterone Signalling, Protein ubiquitination, Unfolded Protein Signalling) whereas after refeeding, differences were linked to thermogenesis, metabolic pathways and cell proliferation (p38 MAPK, ERK/MAPK pathway). In both breeds, transcriptional changes related to the immune response were found after restriction and refeeding.

**Conclusions:**

Altogether, the present study indicates that blood transcriptomics can be a useful tool to study differential genetic response to feed restriction in a dynamic way. The results indicate a differential response of blood gene expression to feed restriction and refeeding between breeds, affecting biological pathways that are in accordance with performance and thermoregulatory results.

**Supplementary Information:**

The online version contains supplementary material available at 10.1186/s12864-023-09381-7.

## Background

During the growing period, pigs may encounter periods of feed restriction due to economic reasons or environmental factors. When facing stressful environmental conditions, such as heat stress, poor sanitary conditions, social stress or disease pressure, pigs reduce their feed intake, leading to feed restriction [1–3]. During these periods of feed restriction, the growing pig must adjust its metabolism to maintain homeostasis through changes in nutrient partitioning between growth and maintenance. The animal responses to feed restriction is highly variable within and between populations and part of this variability may have a genetic basis [4, 5]. Our previous work compared the effect of feed restriction on two contrasted breeds, the Creole (CR) breed, a local breed well adapted to tropical conditions and that has not been submitted to genetic selection, and the Large White breed (LW) that has been selected for high growth performance in optimal conditions [6]. Our results suggested that the CR breed may be more tolerant to feed restriction.

In the context of climate change, there is a crucial need of information on local breeds and on their adaptation to specific environmental conditions, as they constitute genetic resources that are essential to maintain livestock systems diversity and ensure food security [7]. The Creole breed represent the main local breed in the Caribbean region and is constituted from a heterogonous population resulting from successive crossings between Iberians stocks introduced into the West Indies during the 16th century and international breeds introduced thereafter [8] This local breed plays an important role in mixed crop-livestock systems [9] and is characterized by slower growth rate, high fat deposition [10], good meat quality [11] and good adaptation to harsh environmental conditions, including feed restriction [6, 12, 13]. Therefore, the CR breed provides a good model to study the genetic variability in the response to feed restriction in pigs.

Advances in high-throughput technologies such as transcriptomics offer opportunities to better understand complex biological mechanisms and to better characterize local breeds lacking this kind of data. The collection of blood samples is relatively easy compared to other tissues and provides the possibility of sampling the same animal at different time points. It is also a technique that would be easily transferable in breeding schemes. Blood is a circulating connective tissue that interacts continuously with the entire body. Therefore, changes related to injury, disease or nutritional stress occurring within the different tissues of the body may trigger modifications in gene expression in the blood [14]. A recent study on divergent selected lines of pigs showed that the blood transcriptome is relevant to identify biological processes affected by genetic selection and feeding strategies [15].

In the present study, we used whole blood transcriptome analysis to better understand the molecular mechanisms underlying the differential breed response to feed restriction. The objectives of the current study were (1) to compare the transcriptome profile in response to feed restriction and refeeding of two contrasted breeds, LW and CR and (2) to investigate the effect of a moderate feed restriction and refeeding on whole blood transcriptome.RNAseq was performed on blood samples of growing LW and CR pigs at two time points: after 3 weeks of feed restriction and after 3 weeks of refeeding. The data was compared with samples from control animals offered the same diet on an *ad libitum* basis throughout the whole experiment.

## Results

### Climatic characteristics

**Fig. 1:**
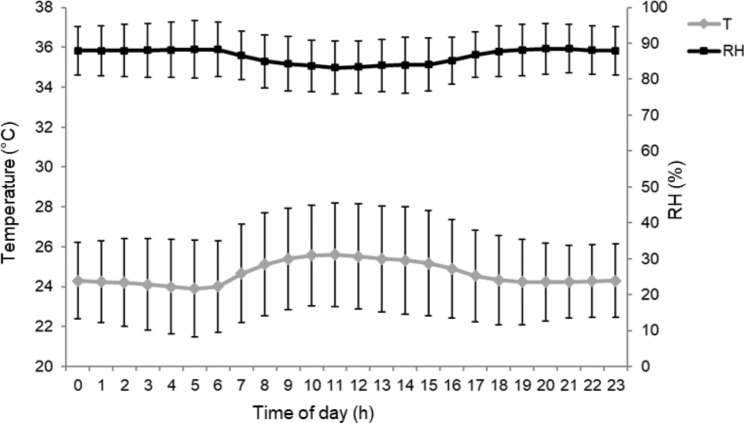
Daily climatic fluctuation of average ambient temperature (T – black line) and average relative humidity (RH – grey line) in the pig building facility. Error bars represent standard deviation

Figure 1 shows the variation of hourly ambient temperature and relative humidity in the experimental facility. The average ambient temperature and relative humidity during the trial were 25.5 °C ± 0.5 °C and 88.4% ± 1.8%, respectively. The hourly fluctuation of ambient temperature showed that the minimum and maximum values were reached at 0600 h (23.9 °C) and at 1200 h (25.6 °C). Relative humidity was greatest at 2100 h and lowest at 1200 h (i.e., 88.5 and 83.2%, respectively).

### Animal performance

Effect of dietary treatment and period on growth performance parameters are presented in Table 1. During feed restriction in Period 2 (P2), growth performance was negatively affected. Irrespective of breed, during P2, Restricted Feeding (RF) pigs had lower average Daily Feed Intake (ADFI) and Average Daily Gain (ADG) than Normal Feeding (NF) pigs (1.71 vs. 1.32 kg/day and 475 g/d vs. 766 g/day, P < 0.01 and P < 0.001, respectively). During P2, RF pigs were also less efficient than NF pigs (0.38 vs. 0.48, P > 0.05). During refeeding, in P3, ADFI and ADG were similar between NF and RF pigs (P = 0.42, P = 0.87 respectively). ADG and Feed Efficiency (FE) for the total experiment period (P1 to P3) were affected by feed restriction (754 vs. 665 g/day and 0.45 vs. 0.38, P < 0.01) but ADFI for the whole experimental period did not differ significantly between treatment (P = 0.77).

**Table 1:** Effect of treatment and period on growth performance

Item	Normal Feeding	Restricted Feeding	RSD^1^	Significant effect^2^
Number of pigs	17	27		
Final BW^3^, kg				
d0	33.9^a^	34.1^a^	0.9	R***, B***, P***, T*, PxT***, PxB***, PxBxT**
P1	37.7^b^	38.5^b^		
P2	54.1^c^	48.2^d^		
P3	69.7^e^	63.9^f^		
ADFI^4^, kg/d				
P1	1.59^a,b^	1.41^b,c^	1.0	R***, B***, P***, PxT***, PxBxT†
P2	1.71^a^	1.32^c^		
P3	1.98^d^	2.13^d^		
total test period	1.81^a^	1.76^a^	1.0	R***, B***
ADG^5^, g/d				
P1	691.9^a^	714.3^a^	1.1	BW_weaning_***, R***, B*, P***, T*, PxT***
P2	766.0^a^	475.0^b^		
P3	765.2^a^	775.3^a^		
total test period	754.1^a^	665.2^b^	1.0	BW_weaning_***, R***, B†, T**
FE^6^, kg of gain/kg of feed				
P1	0.43^a,b,c^	0.53^a^	0.12	R***, B*, PxT†, BxT*
P2	0.48^a^	0.38^b,c^		
P3	0.46^a,c^	0.34^b^		
total test period	0.45^a^	0.38^b^		R***, B**, T**, BxT*

^a−f^ Within a period, means with a different superscript letter differ, P < 0.05

^1^Residual Standard Deviation

^2^From an analysis of variance with a linear mixed model including the effects of Treatment (T), Breed (B), Period (P), Replicate (R) (and Body weight at Weaning for ADG and BW) and their interactions as fixed effect. Statistical significance: ***P < 0.001, **P < 0.01, *P < 0.05, †P ≤ 0.10

^3^BW = Body Weight

^4^ADFI = Average Daily Feed Intake

^5^ADG = Average Daily Gain

^6^FE= Feed Efficiency

**Fig. 2:**
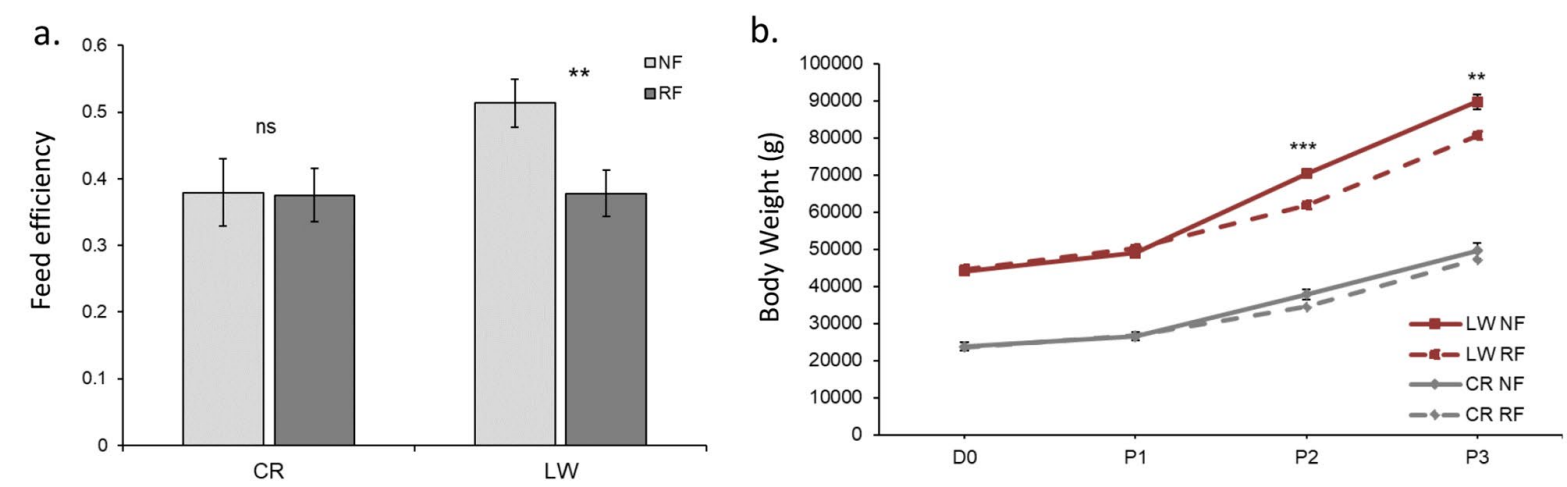
Breed by treatment interactions. (**a**) Feed efficiency over the total experimental period in Creole (CR) and Large White (LW) pigs under Normal Feeding (NF) or Restricted Feeding (RF) treatment. (**b**) Body weight (g) over the 3 experimental periods: P1 (ad libitum for all), P2 (restriction period for the RF group/ ad libitum for the NF group) and P3 (ad libitum for all), starting from day 0 (D0) in Creole (CR, grey) and Large White (LW, red) pigs under Normal Feeding (NF, plain line) or Restricted Feeding (RF, dotted line) treatment. Data are reported as least squares means and error bars represent standard error. Asterisks indicate significant differences between treatments ***P < 0.001, **P < 0.01, *P < 0.05

There was a significant interaction for breed x treatment x period for FE for the whole experimental period (P < 0.05). FE in CR was similar between treatments (0.38 on average, P = 0.90) whereas in LW, FE was lower in RF than in NF (0.51 vs. 0.38 g/d, P < 0.01) (Fig. 2a). We also found significant breed x treatment interaction for Body Weight (BW). In CR, BW at the end of P2 and at the end of P3 did not differ significantly between NF and RF pigs (P = 0.14 and P = 0.48, respectively), whereas in LW, BW was significantly lower in RF pigs after P2 (-12%, P < 0.001), resulting in a lower BW at the end of the experiment (-10% in P3, P < 0.01) (Fig. 2b).

### Thermoregulatory responses

Effect of dietary treatment and period on thermoregulatory responses are presented in Table 2. We found a significant treatment x period interaction for Skin Temperature (ST) and Rectal Temperature (RT) (P < 0.05). During feed restriction in P2, ST and RT were lower in RF pigs than in NF pigs (34.6 °C vs. 35.7 °C, P < 0.001 and 39.1 °C vs. 39.4 °C, P < 0.05, respectively). No effect of breed x treatment was found for ST or RT (P > 0.05). Irrespective of treatment or period, ST was lower in CR pigs compared to LW (35.6 °C vs. 36.4 °C, P < 0.001).

**Table 2:** Effect of treatment and period on thermoregulatory parameters

Item	Normal Feeding	Restricted Feeding	RSD^1^	Significant effect^2^
Number of pigs	17	27		
Rectal Temperature, °C				
P1	39.6^a^	39.5^a^	0.2	R***, P***, PxT**
P2	39.4^a^	39.1^b^		
P3	38.7^c^	39.0^b^		
Skin Temperature, °C				
P1	36.6^a^	36.5^a^	1.0	R*, B**, T*, P***, PxT*
P2	35.7^b^	34.6^c^		
P3	36.3^a,b^	36.3^a,b^		

^a−c^ Within a period, means with a different superscript letter differ, P < 0.05

^1^Residual Standard Deviation

^2^From an analysis of variance with a linear mixed model including the effects of Treatment (T), Breed (B), Period (P), Replicate (R), and their interactions as fixed effect. Statistical significance: ***P < 0.001, **P < 0.01, *P < 0.05, †P ≤ 0.10

### mRNA read alignment and differential gene expression

RNAseq analysis was performed to analyse whole blood transcriptional profile from the 2 dietary treatments and the 2 breeds. An average of approximately 48 millions reads was obtained for each individual sample, which were then assembled and mapped to the annotated *Sus scrofa* 11.1 genome assembly.

**Fig. 3:**
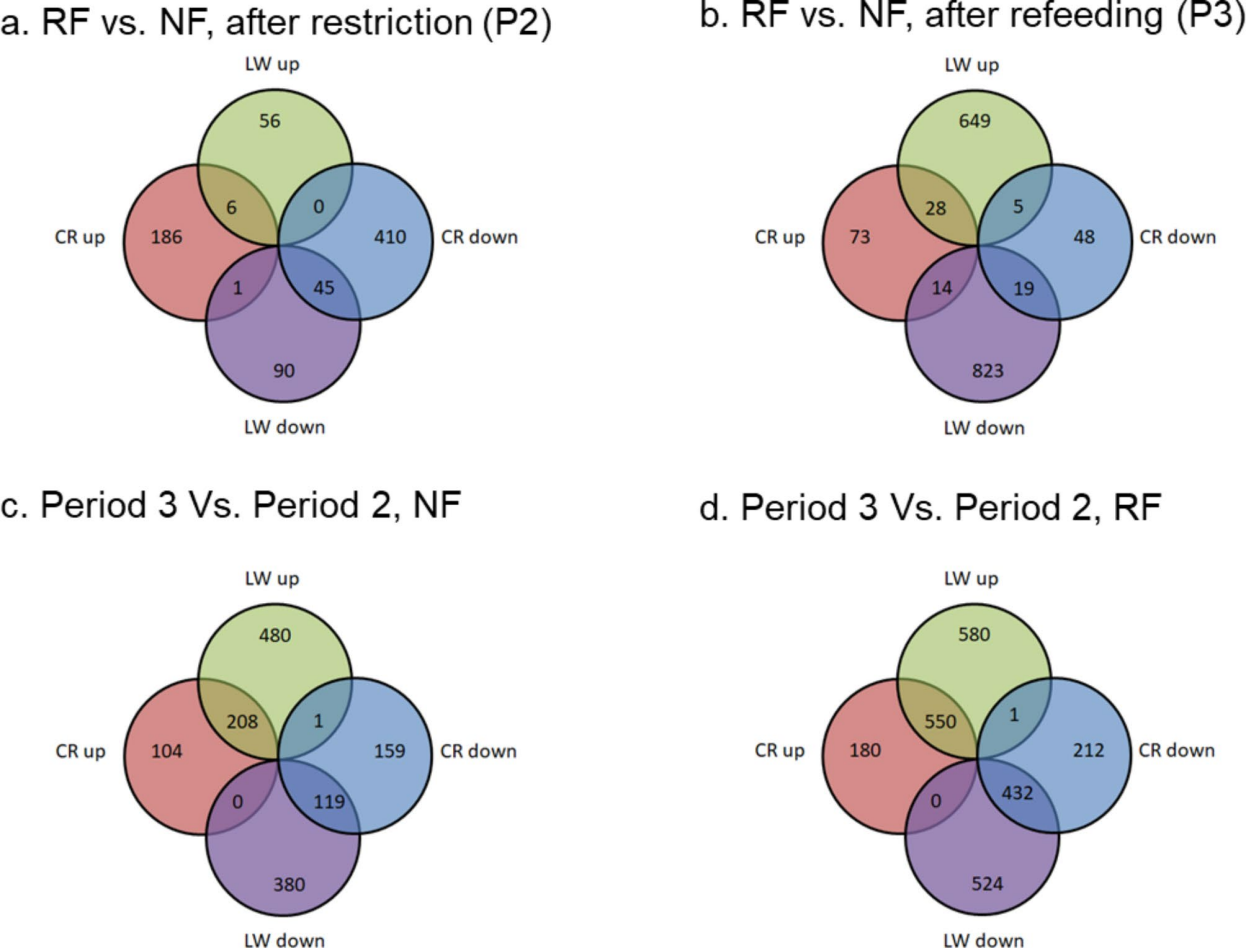
Venn diagrams displaying the number of differentially expressed genes (DEG) in Large White (LW) and Creole (CR) pigs for each comparison. RF: Restricted Feeding, NF: Normal Feeding. P2: restriction period, P3: refeeding period. Numbers in overlapping areas represent DEGs shared by both breeds

The number of Differentially Expressed Genes (DEGs) and log2 fold change in each comparison and breed is shown in Table 3. Following the period of feed restriction, at the end of P2, 648 genes were differentially expressed (DE) in CR, whereas 198 were DE in LW (Fig. 3a). Of the 648 DEG in CR, 193 were up-regulated and 455 down-regulated. In LW, of the 198 DEG, 62 up-regulated and 136 down-regulated. CR and LW shared 51 DEGs in response to feed restriction, with 45 down-regulated and 6 up-regulated.

**Table 3:** Number of differentially expressed genes (DEG) for each comparison

	DEG (FDR < 0.05)	Log 2 fold change range
RF vs. NF, after P2		
LW	198	-1.58 ; 1.12
CR	648	− 5.13 ; 5.00
RF vs. NF, after P3		
LW	1538	− 6.40 ; 3.62
CR	187	− 1.83 ; 2.63
P3 vs. P2, NF		
LW	1188	− 3.98 ; 5.84
CR	591	− 3.48 ; 2.80
P3 vs. P2, RF		
LW	2087	− 3.38 ; 3.30
CR	1375	− 3.02 ; 3.81

FDR : False Discovery Rate

NF : Normal Feeding

RF : Restricted Feeding

P2 : Period 2 (restriction period)

P3 : Period 3 (refeeding period)

Following refeeding, we found a higher number of DEG in LW than CR (1538 in LW vs. 187 in CR) (Fig. 3b). After refeeding, in both breeds, the majority of DEG were up-regulated (55% and 61% upregulated, in LW and CR, respectively) whereas after restriction, DEG were mostly down-regulated (69% and 70% downregulated, in LW and CR, respectively). Few DEG were shared by both breeds, with 28 upregulated and 19 down-regulated. An additional 21 genes were shared by both breeds but the direction of the fold change was reversed between the two breeds.

When the data was analysed within treatment (RF, P3 vs. P2), comparing animals after refeeding to the same animals after restriction, 2087 genes were differentially expressed in LW and 1375 in CR (Fig. 3d). A large proportion of DEG were shared by both breeds (47% of DEG in LW and 71% in CR). Within the NF treatment (Period 3 vs. Period 2), which correspond to the normal growth of the animals, in both breeds, we found fewer DEG than within the RF treatment (1188 vs. 2087 and 591 vs. 1375, for LW and CR respectively) (Fig. 3c).

### Gene ontology and pathway analysis

**Fig. 4:**
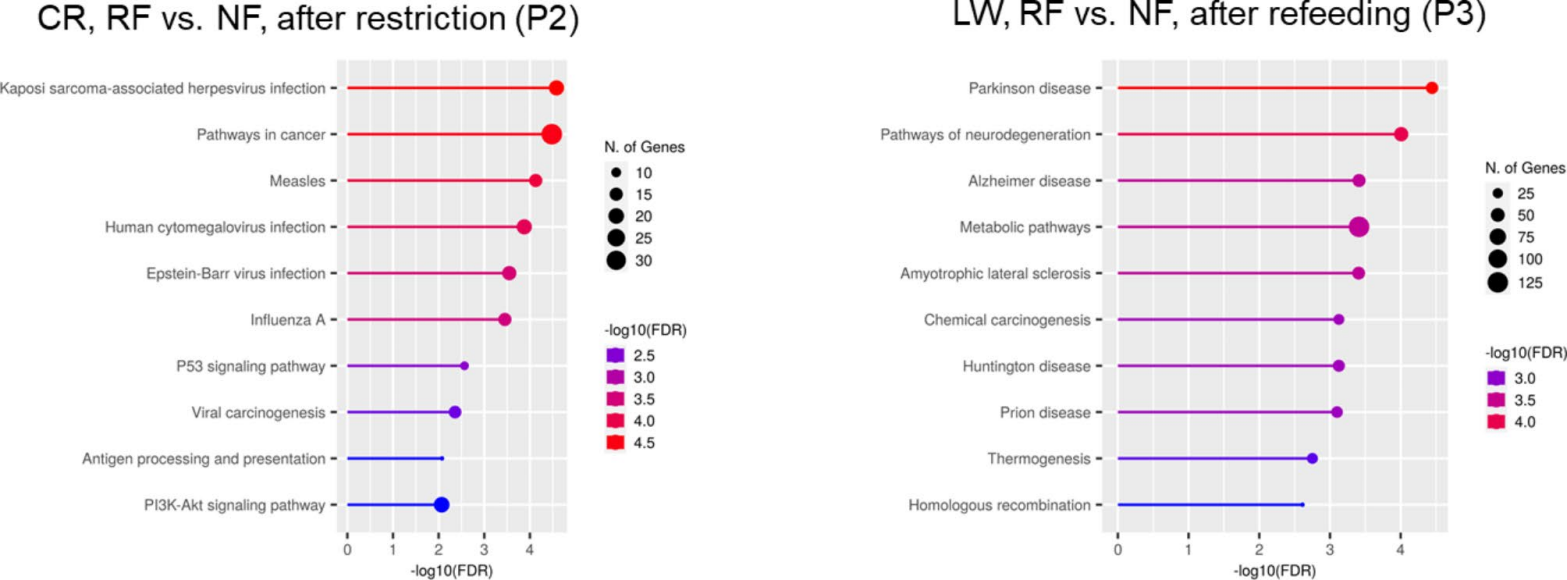
Top 10 significant KEGG pathways identified by ShinyGO [16–18] using DE genes between treatments. CR: Creole, LW: Large White, RF: Restricted Feeding, NF: Normal Feeding

The DEG from each comparison were submitted to ShinyGO [16] for Gene Ontology (GO) analysis. Pathway analysis based on the KEGG database revealed 39 enriched pathways at the end of P2 for CR and 18 at the end of P3 for LW (Top 10 shown in Fig. 4). However, the smaller number of DEG identified at the end of P2 for LW and at the end of P3 for CR did not allow to reach any significant KEGG pathway enrichment.

**Fig. 5:**
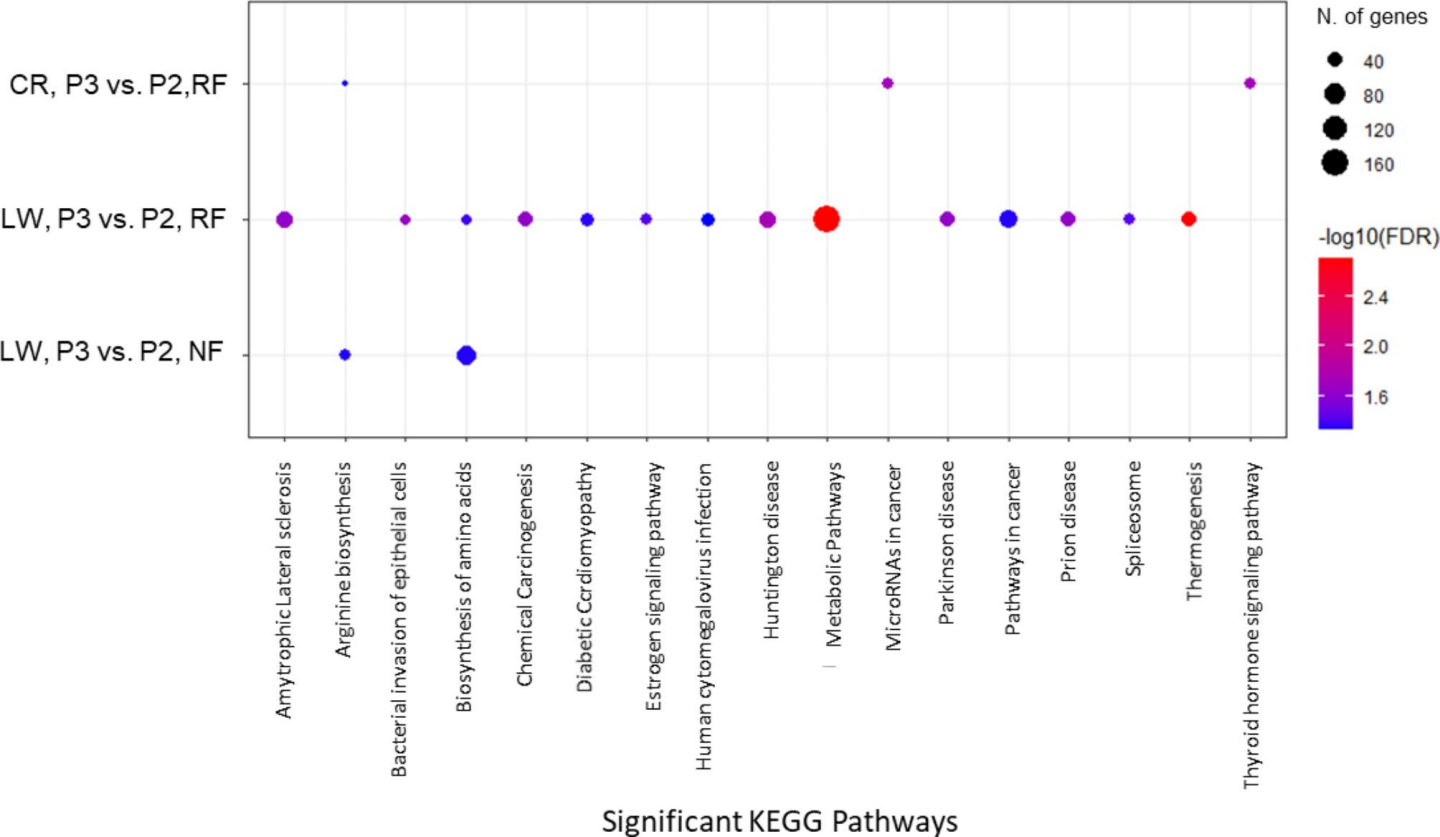
Top 10 significant KEGG pathways identified by ShinyGO using DE genes within treatments. CR: Creole, LW: Large White, RF: Restricted Feeding, NF: Normal Feeding

When analysed within the NF treatment (P3 vs. P2), ShinyGO could not find any KEGG enrichment for CR and only 2 pathways were found for LW. Within the RF treatment (P3 vs. P2), 3 KEGG pathways were enriched in CR and 14 for LW. For LW, the number one enriched pathway within the RF treatment was thermogenesis followed by metabolic pathways. Enriched pathways within treatments are shown in Fig. 5. The 3 comparisons within treatment in which KEGG pathways were significantly enriched had amino acid biosynthesis or arginine biosynthesis pathway enriched.

### Ingenuity pathway analysis (IPA)

After feed restriction, at the end of P2, Ingenuity Pathway Analysis (IPA) identified 29 significant canonical pathways for LW and 179 for CR. Whereas after refeeding, at the end of P3, IPA found 30 canonical pathways for LW and 27 for CR. The top 5 significant pathways for each comparison are shown in Tables 4 and 5 and all the significant pathways and molecules involved are in Supplementary Tables 1 and 2.

**Table 4:** Top 5 significant canonical pathways identified by Ingenuity Pathway Analysis for each comparison in Large White

Comparison	Ingenuity Canonical Pathways	-log(p-value)	Overlap ratio (%)	Z-score
RF vs. NF. after P2	Protein Ubiquitination Pathway	2.82	2.55	
	Unfolded protein response	2.65	4.44	
	Aldosterone Signaling in Epithelial Cells	2.48	3.05	
	Sirtuin Signaling Pathway	2.05	2.05	-2
	SNARE Signaling Pathway	2.02	2.94	0
RF vs. NF. after P3	Oxidative Phosphorylation	3.44	7.21	1.414
	HIF1α Signaling	3.34	5.29	0.302
	Apelin Adipocyte Signaling Pathway	2.51	6.59	-0.816
	HER-2 Signaling in Breast Cancer	2.5	4.41	-1.414
	Mitochondrial Dysfunction	2.25	4.68	
P3 vs. P2. NF	Superpathway of Citrulline Metabolism	3.96	33.3	-0.447
	Citrulline Biosynthesis	3.81	44.4	0
	Sirtuin Signaling Pathway	3.64	7.88	0.5
	EIF2 Signaling	3.51	8.48	-1.387
	Primary Immunodeficiency Signaling	3.19	14.3	
P3 vs. P2. RF	Protein Ubiquitination Pathway	5.55	14.2	
	Sirtuin Signaling Pathway	4.92	13.4	-0.192
	Huntington’s Disease Signaling	4.15	12.7	-1.414
	Inhibition of ARE-Mediated mRNA Degradation Pathway	4	14.9	0.471
	cAMP-mediated signaling	3.59	12.8	0.784

NF : Normal Feeding

RF : Restricted Feeding

P2 : Period 2 (end of restriction)

P3 : Period 3 (end of refeeding)

**Table 5:** Top 5 significant canonical pathways identified by Ingenuity Pathway Analysis for each comparison in Creole

Comparison	Ingenuity Canonical Pathways	-log(p-value)	Overlap ratio (%)	Z-score
RF vs. NF. after P2	Interferon Signaling	7.34	25	-3
	Pancreatic Adenocarcinoma Signaling	4.8	9.52	-0.632
	Ephrin Receptor Signaling	4.51	7.39	-2.309
	RAC Signaling	4.4	8.7	-1.265
	Th1 Pathway	4.23	9.02	-1.667
RF vs. NF. after P3	Citrulline Biosynthesis	2.91	22.2	
	Antigen Presentation Pathway	2.8	7.69	
	Superpathway of Citrulline Metabolism	2.46	13.3	
	Sirtuin Signaling Pathway	2.1	2.05	-0.447
	Role of PKR in Interferon Induction and Antiviral Response	2.06	2.94	0
P3 vs. P2. NF	Interferon Signaling	5.5	19.4	-1.89
	Gαs Signaling	3.34	7.2	0
	Role of Hypercytokinemia/hyperchemokinemia in the Pathogenesis of Influenza	3.03	8.14	-1.134
	Myo-inositol Biosynthesis	2.5	40	
	cAMP-mediated signaling	2.42	4.68	0.302
P3 vs. P2. RF	Protein Ubiquitination Pathway	5.4	10.5	
	Sirtuin Signaling Pathway	5.34	10.3	-0.229
	Virus Entry via Endocytic Pathways	3.45	12.5	
	Role of BRCA1 in DNA Damage Response	3.36	13.8	0
	Aldosterone Signaling in Epithelial Cells	3.34	10.4	-0.707

NF : Normal Feeding

RF : Restricted Feeding

P2 : Period 2 (end of restriction)

P3 : Period 3 (end of refeeding)

**Fig. 6:**
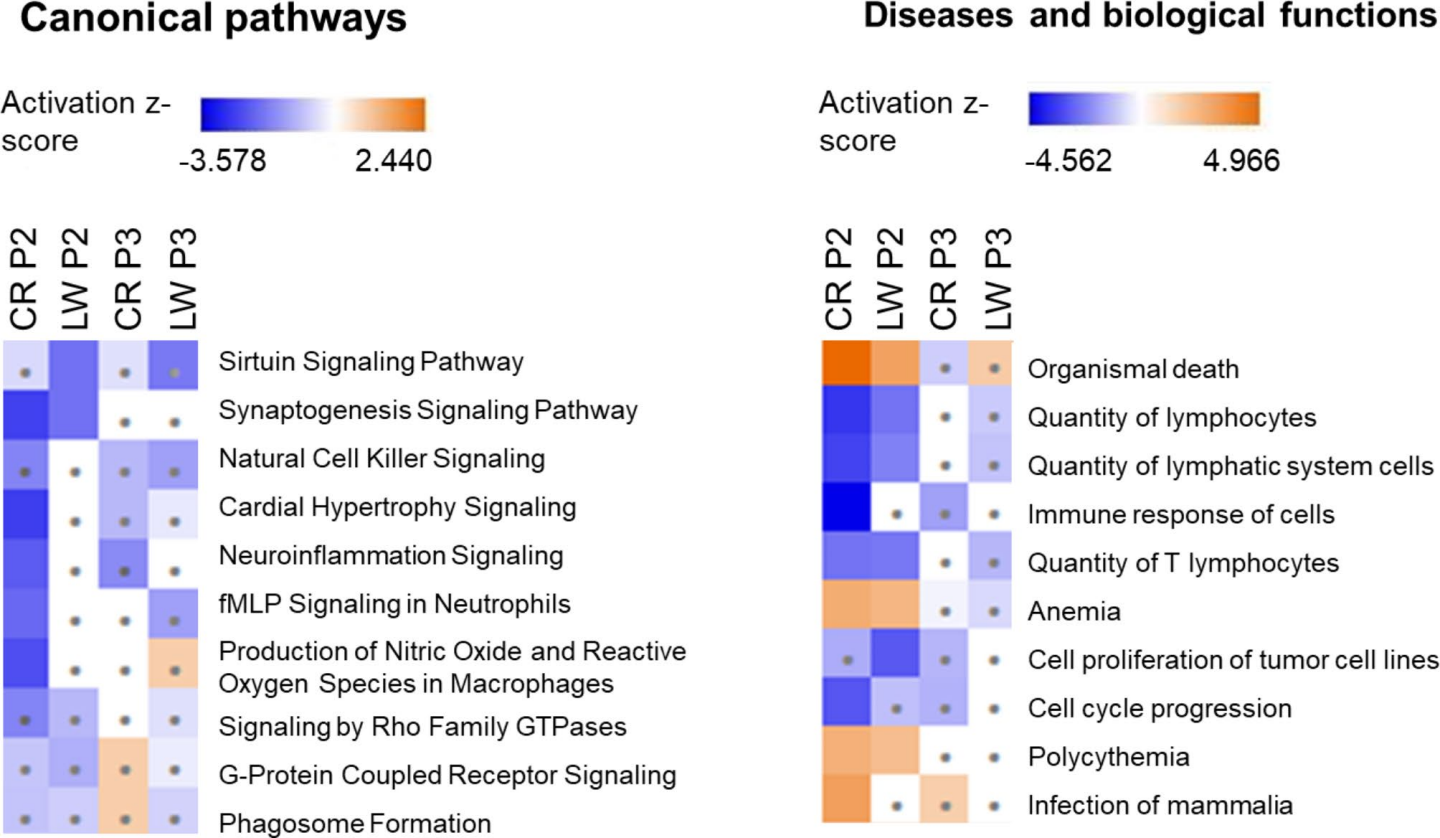
Heat map of canonical pathways and diseases and biological functions identified by Ingenuity Pathway Analysis using DE genes between treatments (RF vs. NF) CR: Creole, LW: Large White, RF: Restricted Feeding, NF: Normal Feeding. P2: restriction period, P3: refeeding period. Squares with dots indicates pathways for which activation/inhibition was not significant (z-score <|2|)

IPA was also used to compare results from the different comparisons in the 2 breeds between treatments (NF vs. RF), over time (after restriction and after refeeding). The top 10 canonical pathways and the top 10 diseases and biological functions were compared (Fig. 6). When comparing the 2 breeds after restriction, synaptogenesis signalling was the only pathway to be significantly inhibited (z-score < -2) in both breeds and it was no longer inhibited after refeeding. In CR, after restriction, enriched pathways were inhibited and mostly related to the immune response (natural cell killer signalling, neuroinflammation signalling, production of nitric oxide). When comparing results after restriction and after refeeding, all pathways and disease and biological functions had a z-score closer to 0 (lower activation) after P3 than after P2. For disease and biological functions, “organismal death”, “anemia”, “polycythemia” were activated in both breeds after restriction but it was no longer the case after refeeding. “Quantity of lymphocytes” was inhibited in both breeds after restriction. After refeeding, “quantity of lymphocytes” was still inhibited in LW to a lower extent but not in CR. “Immune response of cells” was inhibited in CR after restriction and to a lower extent after refeeding.

Intra-treatment analysis (P3 vs. P2) in IPA led to higher enrichment in RF than in NF in both breeds (78 vs. 75 enriched pathways in LW and 118 vs. 51 CR in CR). Overall, Z-score were lower than for the between treatment comparison, with no z-score above |2| (Table 3). Among the top 5 pathways for the intra-treatment comparison, we found: Protein ubiquitination, Aldosterone Signalling in Epithelial cells (P3 vs. P2, RF in CR), and Sirtuin Signalling Pathway (P3 vs. P2, RF and NF in LW and P3 vs. P2, RF in CR) which were also enriched after restriction in LW.

**Fig. 7:**
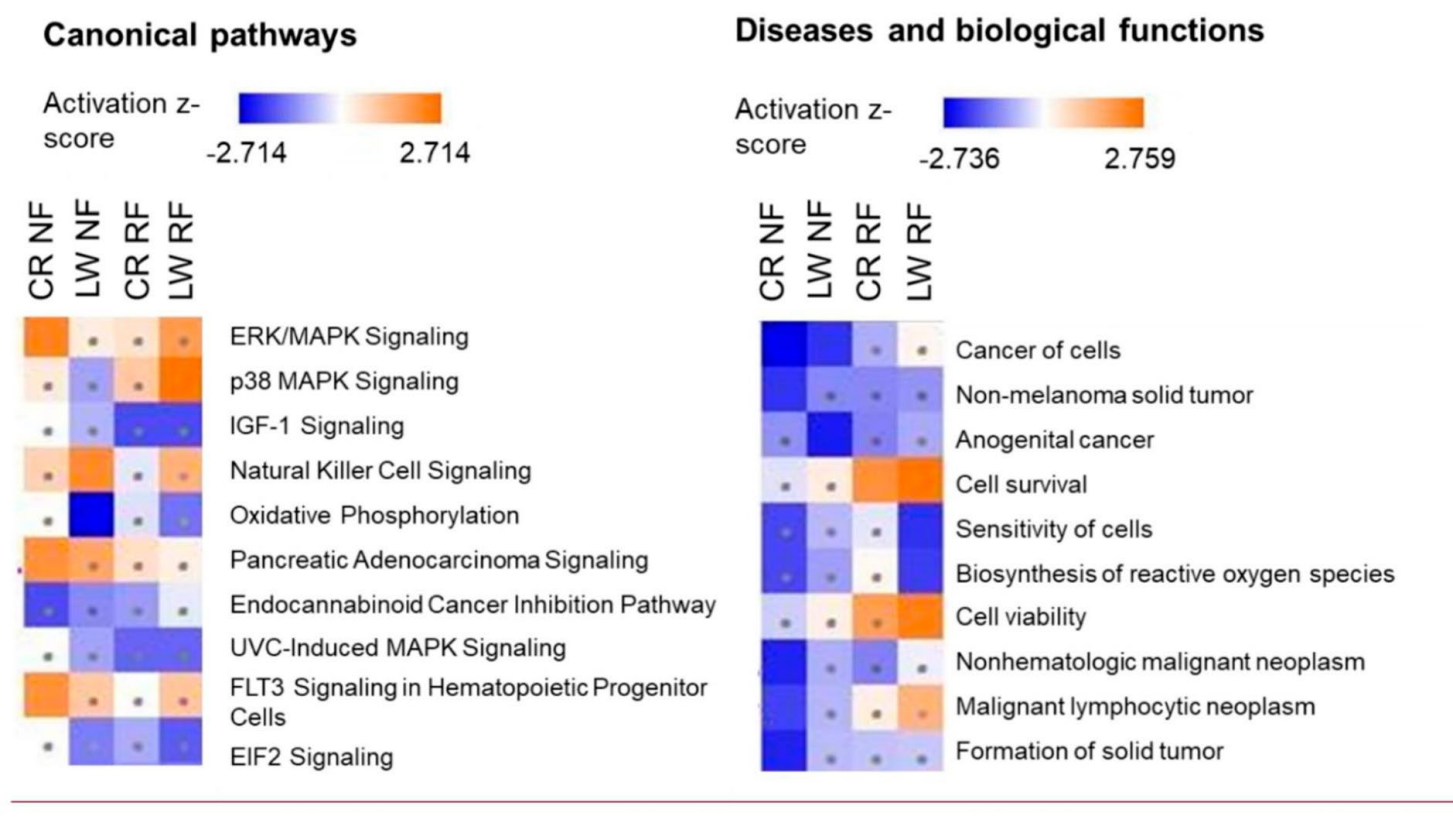
Heat map of canonical pathways and diseases and biological functions identified by Ingenuity Pathway Analysis using DE genes within treatments (P3 vs. P2). CR: Creole, LW: Large White, RF: Restricted Feeding, NF: Normal Feeding. P2: restriction period, P3: refeeding period. Squares with dots indicates pathways for which activation/inhibition was not significant (z-score <|2|)

IPA was used to compare the z-score results from the different intra-treatment (P3 vs. P2) comparisons in the 2 breeds. This comparison helps to visualize differences during the refeeding period between NF and RF and differences in the breed response to refeeding. The Top 10 canonical pathways and diseases and biological functions are shown in Fig. 7. When comparing the NF and RF group, few differences were observed. Among the pathway which expression differ between the RF and NF group (z-score > |2|), p38 MAPK pathway was activated in LW in the RF treatment, but not in NF treatment. We also observed the reverse situation with activation in NF group and not in RF group for ERK/MAPK signalling and pancreatic adenocarcinoma signalling in CR and Natural cell Killer signalling and Oxidative signalling for LW. Concerning disease and biological functions, both in NF and RF and in both breeds, cancer was mainly represented in the top 10 functions, as being inhibited. The main differences observed between NF and RF treatments were “cell survival” and “cell viability” which were activated in both breeds in the RF group but not in the NF treatment.

## Discussion

Periods of feed restriction may occur during pig growth due to economic reasons or external factors, such as heat waves, inflammatory stress, feed transition or social stress [3]. Few studies have investigated the effect of feed restriction and refeeding on livestock transcriptome [19–21] and to our knowledge, there is no comparative analysis of the transcriptomic response to feed restriction and refeeding in different pig breeds. The present study aimed to investigate the effect of feed restriction and refeeding on the blood transcriptome of growing pigs from two contrasted breeds.

***Gene x Environment effects on animal performance*** Performance results were in accordance with previous studies on feed restriction with a reduction of growth performance during the period of reduced feeding [6, 22, 23]. However, in contrast with our previous study [6], in which few Gene x Environment (GxE) effects were found in the response to feed restriction between LW and CR, here, we found significant breed x treatment effects on performance traits. Over the whole experimental period, LW had reduced FE in RF compared to NF, whereas CR had similar FE in the 2 groups. Similarly, in LW, BW in the RF treatment was lower than in the NF group, whereas no difference was found in CR. Together, these results suggest that the CR breed may be more tolerant to feed restriction than LW. This is in line with previous work showing that CR are more tolerant to heat stress than LW [24, 25] and more generally on the positive association between environmental sensitivity and selection for high levels of production [26, 27]. The difference in GxE interactions between our two studies may arise from the difference in the length and severity of the feed restriction, which was shorter and more drastic in our first study (6 days of feed restriction at -50% of ADFI). The mechanisms involved in longer but less severe restriction are different, in particular the relative importance of maintenance over growth is increased in the case of a drastic feed restriction, leading to a reduction of ADG that is greater than the reduction in FI [22]. A moderate but longer feed restriction (as in this experiment) may have allowed to observe more GxE interactions by allowing acclimation processes to occur in the CR breed.

### Differential transcriptomic response to feed restriction in Creole and large White

RNAseq analysis comparing the two feeding groups (RF vs. NF) show that after restriction there were more DEGs in CR than LW, suggesting that the response elicited by feed restriction is stronger in CR than LW. Consequently, after restriction, we also identified more enriched pathways in GO and IPA analysis for CR than LW. Therefore, despite being more tolerant to feed restriction, CR show higher transcriptional response to feed restriction. In the literature, there are several examples of plastic mechanisms resulting in overall robustness of the animal. For instance, protein turn-over, which is involved in the response to various physiological scenarios (maintenance of homeothermy, combating infection, nutritional status…), needs to be highly variable to provide for metabolic regulation and adaptation [27, 28]. Similarly, plasticity in the activity of the hypothalamic-pituary-adrenal gland, which is the most important stress-responsive neuroendocrine system, have been shown to influence several robustness traits positively [29]. Understanding how the plasticity of the transcriptional response in certain genotypes allow for better robustness is therefore essential in the aim to breed for robustness, especially in the context of climate change.

KEGG enrichment showed that the main pathways triggered after feed restriction in CR were related to immunity. Similar results were found after IPA analysis regarding canonical pathways after restriction in CR. The most enriched pathways were related to the immune response and viral infection (Interferon signalling, Th1 pathway), cancer (Pancreas adenocarcinoma signalling, Rac signalling) and Ephrin receptor signalling, which is involved in the maintenance of several processes including angiogenesis, stem cell differentiation and cancer. Finding many genes related to immunity in the blood transcriptome is not surprising as blood cells constitute one of the first lines of immune defence [14]. Similar findings were found in pig studies on blood transcriptome response to genetic selection for feed efficiency and nutritional status [15, 30]. Moreover, genes involved in the immune response were also found to be differentially expressed after dietary restriction in beef cattle jejunal epithelium [19]. Reports in mice, human and rats have also described improved immune function after periods of caloric restriction [31–33]. The main hypothesis is that the immune response may be involved in nutrient partitioning, allowing activation of tissue mobilisation during dietary restriction [34].

GO analysis for LW after restriction comparing RF to NF did not allow to reach any enrichment, probably due to the low number of DEG. Nevertheless, disease and biological functions found with IPA in LW and CR after restriction were mainly related to the immune response (quantity of lymphocytes and T-lymphocytes, immune response of cells). Interestingly, only 3 disease and biological functions were activated after restriction in both breeds, which were “organismal death”, “anemia” and “polycthemia”, suggesting that feed restriction may also trigger genes associated with organismal death and blood defects. The canonical pathway comparison between breeds led to only one common pathway with a z-score < 2 in both LW and CR, which was synaptogenesis. Chronic stress exposure in rats and non-human primates have been shown to induce atrophy of dendrites and decreased glia and neurogenesis in the adult hippocampus [35, 36]. The mechanisms that control food intake also involve communication between gut, adipose tissue and the central nervous system through hormones and peptides circulating in the blood. We could therefore hypothesize that feed restriction generates stressful signals that may affect synaptogenesis.

The Top 5 canonical pathways found with in IPA in the two breeds after restriction did not overlap, suggesting differential response to feed restriction between breeds. In LW, several DEGs in the Top3 enriched pathways found in IPA encodes for Heat Shock Protein (HSPs): DNAJA1, DNAJC17, DNAJC9, HSP90AA1, HSPA12B (Table S1). HSPs are highly conserved proteins playing an essential role in the cellular stress response [37]. The expression of HSP could be linked to the fact that the present experiment takes place in a tropical climate, with a mean temperature of 25.5 °C, which is above growing pig thermoneutral temperature [2]. However, the differential expression of HSP was found comparing RF and NF after restriction, indicating that the response observed is related to the feed diet. Proteomic studies on short-term heat stress (12 h) using pair-feeding controls showed that pigs with a reduced plane of nutrition in thermoneutral conditions had increased HSP70 [38]. HSP are also part of the common over-represented pathways Aldosterone Signaling in Epithelial Cells, Protein ubiquitination pathway and Unfolded Protein Signaling. Aldosterone is the main mineralocorticoid hormone synthetized in the adrenal gland and plays a major role in the control of arterial blood pressure and extracellular volume homeostasis [39]. In rats, aldosterone has been related with an increase of feed intake and weight gain [40]. Genes encoding for HSPs and involved in the aldosterone pathway have been identified as over-expressed in the liver and duodenum of pigs with low FE compared to high FE pigs [41]. Similar findings were observed in the spleen and small intestine transcriptome of beef steers phenotypically divergent for feed intake and body weight gain [42, 43]. The authors found that multiple HSP proteins were associated with low gain/low intake beef steers. Interestingly here, upregulation of HSP after feed restriction is detected in LW and not in CR, indicating that despite the lower ADG of CR, HSP are not triggered upon feed restriction in that breed. This evidence suggest that LW have higher stress response than CR, which is supported by the performance results obtained and previous studies comparing LW and CR [44]. This results is also in accordance with the positive association between environmental sensitivity and selection for high levels of production [26, 27]. In line with these results, a study comparing HSP90 mRNA expression levels after heat stress in peripheral blood mononuclear cells of LW and CR found an increase of HSP90 mRNA expression in both breeds after 6 h, but a significant decrease in CR pigs after 9 h [45]. The authors suggested that the difference observed after 9 h could be due to a reduced impact of heat stress on protein conformations in CR pigs.

The Sirtuin Signalling Pathway was enriched after restriction in LW. Sirtuins are NAD^+^-dependent histone and protein deacetylases, which play an important role in the regulation of energy homeostasis in mammals but also in aging, cancer, inflammation DNA repair and cellular response to stress [46]. In mice, sirtuins are upregulated upon fasting and caloric restriction in brain, muscle and fat [47]. In pigs fed a high fiber diet, Sirt1 expression was increased in colonic tissues compared to the low fiber diet, indicating an effect of the diet on Sirt1 expression [48]. In the present study, sirtuin signalling pathway was predicted to be inhibited by IPA (z-score = 2) only in LW after restriction, which contrasts with literature on sirtuins which are reported to be activated upon caloric restriction in different species. When inspecting the DE molecules involved in the Sirtuin signalling pathway after restriction in LW, ATG14, an essential autophagy-specific regulator, was up-regulated. Overexpression of ATG14 activates autophagy in mammalian cells even in nutrient-rich conditions [49]. Sirtuins also modulate autophagy through complex interactions and signalling mechanisms that are not yet entirely elucidated [50]. The response to feed restriction in LW may therefore involve sirtuin signalling and autophagy but remains to be deciphered.

### Differential transcriptomic response to refeeding in Creole and Large White

After refeeding, the number of DEGs was higher in LW than CR, suggesting stronger response to refeeding in LW than CR. In LW, the KEGG pathways identified after refeeding were related to the immune response but also to thermogenesis. Thermogenesis could be triggered during refeeding due to increased feed intake compared to the restriction period, which may generate increased metabolic heat [51]. Thermogenesis was not identified in CR, suggesting differences in metabolism and thermoregulation between the two breeds probably related to their breeding background. The immune response has also been shown to be triggered upon refeeding in beef cattle jejunum transcriptomic profile and could allow more dietary derived energy to be partitioned towards growth during re-alimentation [19]. Interestingly, we did not observe the reversal of the biological mechanisms occurring during restriction as observed in previous studies in beef cattle [19]. The feed restriction being moderate, we could hypothesize that the reversal of the biological mechanisms happened quickly after the beginning of refeeding and that the RNAseq analysis after 3 weeks of refeeding did not allow to observe this process in terms of DEGs.

Despite the greater number of DEGs found in LW than CR after refeeding, the difference in terms of performance between the 2 breeds after refeeding were not significant. In none of the breeds do we observe compensatory growth, i.e. a period of accelerated growth following periods of feed restriction, during refeeding. Compensatory growth in pigs depends on the onset, severity and duration of the restriction period and the onset and duration of refeeding [22, 23, 52, 53]. In the present study, despite a long period of feed restriction and refeeding, the severity of the feed restriction was probably not sufficient to induce compensatory growth. Consistent with this result, pathways and disease and biological functions enrichment in IPA after refeeding led to lower z-score than after restriction, suggesting lower response for both breeds after refeeding than after restriction. Nevertheless, when the RNAseq analysis was performed within treatment across time (P3 vs. P2), the number of DEGs obtained in RF was more than twice the number obtained in NF treatment, suggesting that the NF group analysis overtime corresponds to normal growth whereas the RF treatment analysis corresponds to normal growth plus the effect of refeeding after restriction. In both breeds, pathways enriched in the RF treatment include pathways related to the immune response, to the biosynthesis of amino acids and for LW, to thermogenesis. The biosynthesis of AA is also enriched in the NF group, suggesting that it is not specific to refeeding but more likely required for normal growth of animals. The fact that thermogenesis is also enriched in this comparison confirm that it is likely related to increased feed intake in LW during refeeding. This is consistent with rectal temperature measures that were higher in RF than NF after refeeding, irrespective of breed.

Similar to the results after restriction in LW, protein ubiquitination pathway was also enriched in the intra-treatment comparison, within the RF treatment (P3 vs. P2) in both breeds and aldosterone was enriched in the same comparison but in CR only. The molecules involved were not the same as the ones found in LW after restriction but also involved several HSPs (HSP90AB1, HSP90B1 and several DNAJ). As mentioned before, these molecules have been found to be related to feed intake, ADG and FE [40–43]. HSP expression may also be required for cells to recover from metabolic insults [54]. Therefore, it would be consistent to find this set of molecules DE during refeeding in the RF group. The fact that these pathways are enriched in both breeds suggest that this response to refeeding might be conserved between the two breeds.

The comparative intra-treatment (P3 vs. P2) analysis in IPA for canonical pathways led to few significant enriched pathways. The comparative analysis of IPA did not allow to find pathways that were significantly activated (z-score > |2|) shared by both breeds in RF or NF, suggesting different responses during refeeding but also during normal growth. Among the pathways with significant z-score, p38 MAPK signalling was activated in RF in LW. p38 MAPK is a MAP Kinase involved in the stress response, apoptosis but also cell differentiation and proliferation [55]. The fact that p38 MAPK is activated in LW and not in CR after refeeding could suggest a stronger response to stress or a higher rate of cell proliferation in LW, which would be in line the breeding background of LW focused on production. The comparison for disease and biological function showed that in both breeds, “cell survival” and “cell viability” were activated in RF treatment but not in NF, showing that after refeeding, cell survival and viability are enhanced compared to the restriction period. On the contrary, “cancer of cells” was inhibited in both breeds in NF but not in RF, suggesting that this pathway could be inhibited during normal growth but that is not the case during refeeding.

### Conclusions

In conclusion, the present study indicates that blood transcriptomics can be a useful tool to study differential genetic response to feed restriction in a dynamic way throughout the different periods of stress of the animal life. In both breeds, major transcriptional changes after restriction and refeeding were related to the immune response. Nevertheless, the transcriptional response to feed restriction and refeeding between CR and LW was contrasted both in terms of number of DEGS and enriched pathways. CR demonstrated a stronger transcriptional response to feed restriction whereas LW had a stronger response to refeeding. Most differences in the transcriptional response to feed restriction between CR and LW were related to cell stress response, whereas after refeeding, differences were linked to thermogenesis, metabolic pathways and cell proliferation. In terms of performance, CR were more tolerant than LW to feed restriction regarding both BW and FE, probably due to the strong breeding selection pressure on production of the LW. Therefore, the increased transcriptional response could be responsible for the robustness observed at the performance level. Additional research on local breeds and potential structural variants that could increase the transcriptional response to feed restriction while maintaining performance would contribute to deepening our understanding of post-absorptive metabolism differences between breeds.

## Methods

### Animals and experiment design

A total of 46 growing pigs (23 LW and 23 CR) with an average BW of 35.2 ± 1.2 kg for LW and 17.7 ± 1.6 kg for CR, were used in 2 replicates for the experiment in the semi-open front building of the INRAE experimental farm located in Guadeloupe, French West Indies. The climatic environment followed the tropical climate ambient temperature and humidity. The 2 replicates were carried out during the warm season (November to April) leading to mild ambient temperatures (25.5 °C ± 0.5 °C). The experiment was set during the growing period, from 14 to 23 weeks of age. At 12 weeks of age, pigs were allotted to 2 or 3 pens (5.7 × 2.7 m) with a density of 8–10 pigs/pen (4–5 LW and 4–5 CR) and evaluated after 14 days of adaptation to the new environment. Each pen was balanced in terms of breed (½ LW, ½ CR) and sex within in each breed (½ females, ½ castrated males). Each pen was equipped with a single place electronic feeder (ACEMA 128, ACEMO, Pontivy, France) to record individual feed intake.

**Fig. 8:**
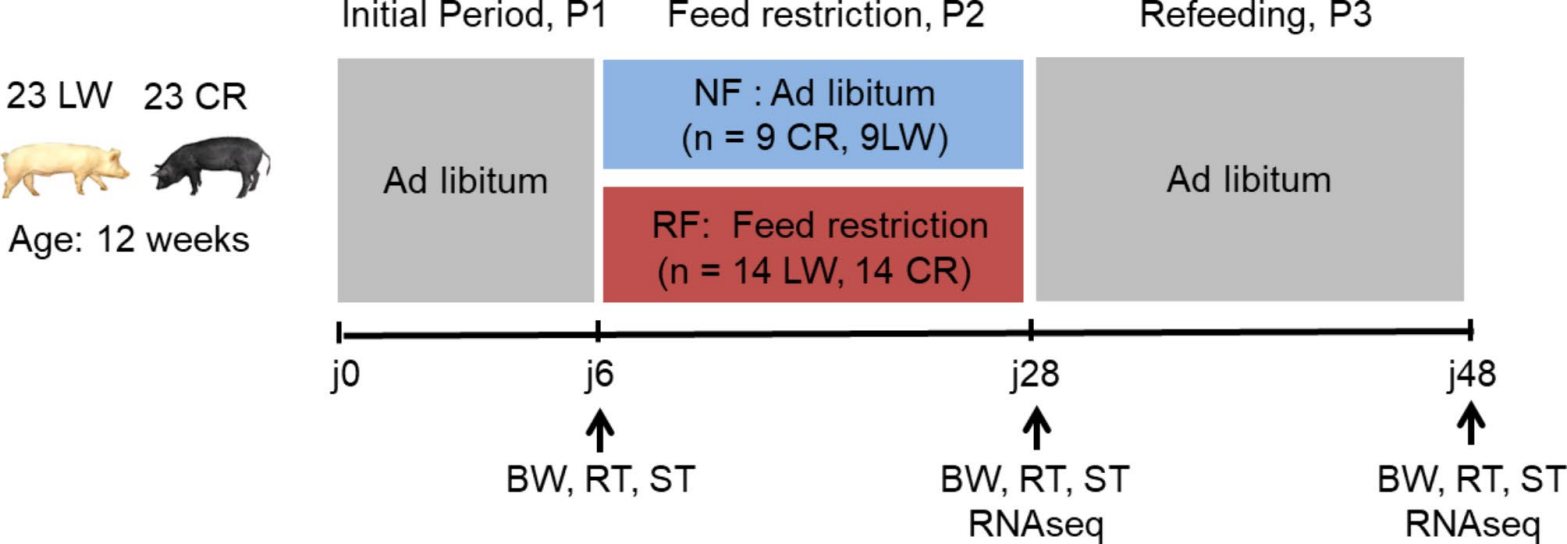
Experimental design. CR: Creole, LW: Large White, RF: Restricted Feeding, NF: Normal Feeding. P2: restriction period, P3: refeeding period, BW: Body Weight, RT: Rectal Temperature, ST: Skin Temperature

Animals were fed a conventional diet as pellet, formulated to meet the nutritional requirements of LW growing pigs according to standard recommendations [56], with corn, wheat middling, and soybean meal, and containing 13.53 MJ of Digestible Energy, 164 g of Crude Protein (Table 6). The experiment consisted of three consecutive periods (Fig. 8). Period 1 (**P1**) was the initial period (7 days) where all pigs were fed *ad-libitum*. Period 2 (**P2**) was a 3-week period during which feed restriction was imposed to specific pens. Due to experimental limitations, the two feeding treatments were not balanced in number of animals. During the first replicate, one pen (referred to as NF, 5 LW and 5 CR) continued to be fed *ad libitum* in P2, whereas 2 pens (referred to as RF, 10 LW and 10 CR) had restricted access to the automatic feeder (from 7:00 to 17:00). For the second replicate, only 2 pens were used, one pen was fed *ad libitum* (4 LW, 4 CR) and the other was feed-restricted (4 LW, 4 CR). Period 3 (**P3**) constituted the following 3-week period and corresponded to the refeeding period during which all animals were fed ad libitum. All pigs had free access to water at all times from a nipple drinker designed to avoid water spillage. A time-restricted feed restriction was chosen to induce a mild restriction but to allow reduced competition that could lead to excessive feeding by some pigs.

During the experiment, 2 CR pigs (one NF, one RF) died due to illness or injury and their data were removed from the database, thus the final dataset consisted of data from 44 pigs [17 NF (8CR, 9LW) and 27 RF (14 LW, 13 CR)].

**Table 6:** Feed composition

Item	
Analysed chemical composition (% of DM)	
Dry matter	88.0 ± 0.4
CP	16.4 ± 0.5
Ash	6.0 ± 0.3
Crude fibre	4.3 ± 0.3
Neutral Detergent Fiber (NDF)^1^	14.8
Fat	3.8
Starch	42.7
Energy value (MJ/kg)^2^	
Gross energy	16.33
DE	13.53

^1^ The samples were pooled for the determination of NDF

^2^ Values calculated according to Le Goff and Noblet (2001) [57]

### Measurements

Ambient temperature and relative humidity were continuously recorded (1 measurement every 15 min) in the building using a stand-alone USB data logger (EL-USB-2+; DATAQ Instruments, Inc., Akron, OH) located near the growing pens. Body weight was measured at the beginning of the experiment (d0) and then at the end of each period (week 15, week 18, week 21).

In order to evaluate the effect of feed restriction and breed on thermoregulation, rectal temperature (**RT**) and skin temperature (**ST**) were measured in all pigs at the end of each period (week 15, week 18, week 21), in the morning between 08:00 and 09:00. Rectal temperature was recorded with a digital thermometer (Microlife Corp., Paris, France) and ST was measured at the shoulder, mid back (P2 site) using a skin surface thermocouple probe (type K, model 88,002 K-IEC; Omega Engineering Inc., Stamford, CT) connected to a microprocessor- based handheld thermometer (model HH-21; Omega Engineering Inc.).

Blood samples were collected at the end of P2 (week 15) and at the end of P3 (week 21) at 08:00 in the morning. Jugular vein blood was obtained (10-mL BD K_2_ EDTA Vacutainers tubes (BD, Franklin Lakes, NJ)) via venepuncture. For samples dedicated to RNA extraction, one volume of blood sample was mixed with one volume of lysis buffer from the Nucleospin RNA blood kit (Macherey-Nagel, Lyon, France). The obtained mixture was then stored at -80 °C for later analyses.

### RNA extraction and quality analysis

Total RNA was extracted from frozen blood samples of 28 animals from the first replicate [9 NF (4 CR, 5LW) and 19 RF (10 LW, 9 CR)] using the NucleoSpin RNA isolation kit (Macherey-Nagel, Hoerdt, France) in accordance with the manufacturer’s instructions. The total RNA concentration was measured with NanoDrop 2000 (ThermoScientific TM, France) and the quality was quantified using an Agilent 2100 Bioanalyzer (Agilent Technologies, France). The extracted total RNA was stored at -80˚C until use.

### Library preparation and sequencing

Library preparation was performed according to Aboshady et al. [58]. High-quality RNA (RIN > 7.5) was used for the preparation of cDNA libraries according to Illumina’s protocols (Illumina TruSeq RNA sample prep kit for mRNA analysis). Briefly, poly-A mRNA was purified from 4 µg of total RNA, fragmented and randomly primed for reverse transcription to generate double stranded cDNA. The cDNA fragments were then subjected to an end repair process, consisting of the addition of a single ‘A’ base, and the ligation of indexed Illumina adapters at both ends of cDNA. These products were then purified and enriched by PCR to create the final bar-coded cDNA library. After quality control and quantification, cDNA libraries were sequenced on 2 lanes on the NovaSeq6000 S4 (Illumina® NEB, USA) to obtain approximatively 48 million reads (100 bp paired-end) for each sample.

### Quality control and read mapping to the reference genome

The quality control check on raw reads in FASTQ format were processed using FASTQC and the Q30, GC content and length distribution of the clean data were calculated. The sequences obtained by RNA-Seq were splice-aligned for each library, using STAR (version 2.3.0e with standard parameters) [59]. The reads were mapped to the *Sus Scrofa* genome (assembly 11.1). HTSeq (http://pypi.python.org/pypi/HTSeq) [60] was used to calculate the number of sequence reads aligned to all protein-coding genes from the ENSEMBL v74 annotation of the *Sus scrofa* genome. The Bioconductor package DeSeq2 [61] was then used to identify differentially expressed genes (DEGs). Four treatment comparisons were tested for DEGs for each breed: (i) RF v. NF at the end of Period 2; (ii) RF v. NF at the end of Period 3; (iii) NF Period 2 v. RF Period 3; and (iv) RF Period 2 v. RF Period 3. Statistically significant (P < 0.05) DEGs with a Benjamini-Hochberg false discovery rate of < 0.05 were deemed to be significant. Analysis of canonical pathways and regulatory effects as well as network analysis were performed using Ingenuity pathway analysis (IPA) software (Ingenuity Systems, Redwood City, CA) for DEGs in each comparison. IPA identifies known regulators, including genes and other molecules that may affect the expression of DE genes, then it calculates a z-score, which is a statistical measure of the match between the expected relationship direction between the regulator and its targets, and the observed gene expression [62]. Moreover, KEGG pathway and Gene Ontology enrichment analyses were performed using ShinyGO [16].

### Calculations and statistical analysis

ADFI was calculated from data collected by the electronic feed dispensers by averaging daily feed intake records for each pig. Average daily feed intake, average daily gain (**ADG**, g/day) and Feed Efficiency (**FE**, kg of gain per kg of feed) were calculated for each period.

Data were analysed using the MIXED procedure of SAS (SAS Inst., Inc., Cary, NC, USA) including the fixed effects of replicate, breed, sex, period, dietary treatment and their interaction. For BW and ADG, BW at weaning was also included as a fixed effect to take into account environmental maternal effect. In all statistical analyses using the MIXED procedure of SAS, the repeated measurements option was used to account for animal effect over time with an unstructured covariance structure, except for thermoregulatory variables for which a compound symmetry covariance structure was used, because of no convergence with unstructured covariance structure. Data are reported as least squares means ± SEM and are considered significant if P < 0.05.

## Electronic supplementary material

Below is the link to the electronic supplementary material.

Supplementary Material 1

Supplementary Material 2

## Data Availability

The datasets generated and analysed during the current study are available in the Sequence Read Archive (SRA) from the NCBI repository, under the following reviewer link: https://dataview.ncbi.nlm.nih.gov/object/PRJNA946175?reviewer=mt3bjhtkaolts45ia9cu1a34t8.
